# Patient characteristics associated with treatment initiation among paediatric patients with Attention-Deficit/Hyperactivity Disorder symptoms in a naturalistic setting in Central Europe and East Asia

**DOI:** 10.1186/s12888-014-0304-x

**Published:** 2014-10-30

**Authors:** Jihyung Hong, Diego Novick, Tamás Treuer, William Montgomery, Virginia S Haynes, Shenghu Wu, Josep Maria Haro

**Affiliations:** Eli Lilly and Company, Windlesham, Surrey, UK; Eli Lilly and Company, Neuroscience Research, Budapest, Hungary; Eli Lilly Australia Pty Ltd, West Ryde, Australia; Eli Lilly and Company, Indianapolis, IN USA; Eli Lilly China, Shanghai, China; Parc Sanitari Sant Joan de Déu, Fundació Sant Joan de Déu, CIBERSAM, Universitat de Barcelona, Barcelona, Spain

**Keywords:** ADHD, Treatment initiation, Patient characteristics, Central Europe, East Asia

## Abstract

**Background:**

Cultural views of Attention-Deficit/Hyperactivity Disorder (ADHD), differing healthcare systems and funding mechanisms, and the availability of mental health services can greatly influence the perceptions, diagnosis, and treatment of ADHD. There is, however, lack of information about treatment practice and the treatment decision-making process for ADHD, particularly in non-Western countries. Our study compared characteristics of paediatric patients newly diagnosed with ADHD symptoms who did and who did not initiate treatment, and also examined whether any differences varied by region in Central Europe and East Asia.

**Methods:**

Data were taken from a 1-year prospective, observational study that included 1,068 paediatric patients newly diagnosed with ADHD symptoms. Clinical severity was measured using the Clinical Global Impression-ADHD-Severity (CGI-ADHD-S) scale and the Child Symptom Inventory-4 (CSI-4) checklist. Logistic regression was used to explore patient characteristics associated with treatment initiation (pharmacotherapy and/or psychotherapy) at baseline for each region.

**Results:**

A total of 74.3% of patients initiated treatment at baseline (78.3% in Central Europe and 69.9% in East Asia). Of these, 48.8% started with both pharmacotherapy and psychotherapy in Central Europe, and only 17.1% did so in East Asia. The level of clinical severity was highest in the combination treatment group in Central Europe, but was highest in the psychotherapy only group in East Asia. In East Asia, treatment initiation was associated with being older, being male, and having a higher CGI-ADHD-S score. In Central Europe, treatment initiation was associated with parental psychological distress, having a higher CSI-4 score, and not being involved in bullying.

**Conclusions:**

Although factors associated with treatment initiation differed to some extent between Central Europe and East Asia, clinical severity appeared to be one of the most important determinants of treatment initiation in both regions. However, the choice between pharmacotherapy and psychotherapy, either alone or in combination, varied substantially across the regions.

**Electronic supplementary material:**

The online version of this article (doi:10.1186/s12888-014-0304-x) contains supplementary material, which is available to authorized users.

## Background

Attention-Deficit/Hyperactivity Disorder (ADHD) is a neurobehavioural condition, characterised by the core symptoms of inattention and/or hyperactivity/impulsivity [1]. It is among the most prevalent mental disorders in childhood and adolescence across countries and regions [2]. Although data on the prevalence of ADHD in non-Western countries (e.g., Central Europe and East Asia) are limited, the available evidence suggests that prevalence rates in these regions are similar to those in Western countries, ranging from 7.5% to 12% among school-aged children and adolescents [3-7].

Treatment for ADHD typically involves both pharmacotherapy and psychoeducational/behavioural treatments, either alone or in combination [8-10]. Several international guidelines on ADHD support the use of these treatment options, although a discrepancy exists between the U.S. [10] and European guidelines in the treatment preferences for first-line therapy [8,11]. For example, the U.S. guidelines developed by the American Academy of Paediatrics recommend the first-line use of pharmacotherapy, preferably combined with behavioural therapies, for school-aged children and adolescents with ADHD, but only behavioural therapies for preschool-aged children [10], whereas the European guidelines developed by the European Network for Hyperkinetic Disorders and the UK guideline developed by the National Institute for Health and Care Excellence recommend that the use of drug treatments be reserved only for those with severe symptoms and impairments or when patients fail to respond to an initial trial of psychological treatments [8,11]. Although European guidelines are, in general, more conservative about the use of pharmacotherapy as a first-line therapy for ADHD due to concerns over potential adverse events during treatment with ADHD medications (albeit rare and mostly manageable [12]), these guidelines consider both pharmacotherapy and psychotherapy as important treatment options for ADHD and suggest that patient age and clinical severity are the most important clinical factors to consider when making treatment decisions for ADHD.

The limited available evidence on the factors associated with treatment initiation is largely consistent with these international guidelines [13,14]. Using data from a 2-year observational study in Europe, Falissard *et al*. found that the initiation of pharmacotherapy at the baseline visit was associated with more severe ADHD symptoms and impairments [13]; however, the study was primarily descriptive and did not examine the associations with other factors. The only study that has explicitly explored patient characteristics associated with the initiation of ADHD treatment was a study by Chen *et al*. based in the United States [14]. While they analysed the initiation of medication among patients with a new ADHD episode using Florida Medicaid claims data, the study was unable to assess the impact of clinical severity on treatment decision due to data constraints and also focused more on the choice between ADHD medications and other psychiatric medications in the treatment of ADHD. The study reported that the initiation of ADHD medications was associated with being older and being male, as well as other patient characteristics including race (White), rural dwelling, foster care, and the diagnosis by primary care physicians [14].

Although patient age and clinical severity are likely to play important roles in the treatment decision-making process for ADHD, a recent study based on the opinions of and feedback from international leaders in this area confirmed the great deal of variation in ADHD diagnosis and treatment practices across and within countries [15]. Cultural views on the disorder, differing healthcare systems and funding mechanisms, and the availability of mental health services were assumed to greatly influence perceptions, diagnosis, and treatment of ADHD. For example, the study indicated that as in the United States, Canadian health professionals tend to view “ADHD as an impairing, often life-long disorder that requires careful assessment and multimodal intervention” [15]. In addition, a wide range of pharmacological and psychosocial interventions are made accessible to the general population through the nationalised healthcare system. Meanwhile, ADHD is still greatly underdiagnosed and undertreated in many parts of the world due to the high levels of stigma attached to mental illness, lack of acceptance of ADHD as a disorder, limited access to services, lack of training in the treatment of ADHD among medical and mental health professionals, and the high acceptance of herbal treatments and homeopathy in some cultures. Although there is lack of empirical evidence on treatment practices for ADHD in non-Western countries, the role of clinical severity in the treatment decision-making process may be greater in these regions because the stigma attached to mental illness and the preference for homeopathic and herbal remedies are likely stronger, thereby the use of psychiatric treatments/medications is likely less accepted in these regions.

Using data from a 1-year, prospective, observational study involving 1,068 paediatric patients newly diagnosed with ADHD symptoms from Central Europe and East Asia, this study compared the demographic and clinical characteristics of those patients who did and did not initiate treatment in routine clinical practice in Central Europe and East Asia and examined whether any differences between them varied by region.

## Methods

### Study design and patient sample

This was a 12-month, international, prospective, non-interventional, observational study primarily designed to examine the treatment patterns and health outcomes among paediatric patients newly diagnosed with ADHD symptoms in actual clinical practice. From October 2005 to July 2006, fifty-eight psychiatrists and paediatricians enrolled a total of 1,068 paediatric patients from eight countries across Central Europe and East Asia (China [n = 301], the Czech Republic [n = 50], Hungary [n = 96], Romania [n = 82], Slovakia [n = 55], South Korea [n = 100], Taiwan [n = 101], and Turkey [n = 283]). This study followed the ethical standards of responsible local committees and regulations from the participating countries, was conducted in accordance with the ethical principles of the Declaration of Helsinki, and is consistent with good clinical practice when applicable to a study of this nature. Ethical Review Board (ERB) approval was obtained wherever required by local law for observational studies (see Additional file 1). The parents/guardians of all patients provided written informed consent, and the patients provided assent. Further details on the study design have been reported elsewhere [16-20].

Child and adolescent outpatients aged 6 to 17 years could participate in the study if they presented within the normal course of care with ADHD symptoms and had not been previously diagnosed with or treated for ADHD. The diagnosis of ADHD symptoms was made by an investigator using standard diagnostic criteria (*Diagnostic and Statistical Manual of Mental Disorders*-4th edition-text revision [DSM-IV-TR] [21] or International Classification of Diseases-10 [ICD-10] [22]). That is, in the clinical judgment of the investigator, at baseline the participating patients were required to have hyperactive, inattentive, or impulsive symptoms or problems associated with ADHD as described in the DSM-IV-TR or hyperkinetic disorders, disturbance of activity and attention, or hyperkinetic conduct disorder as described in the ICD-10. The study excluded those patients who had severe mental retardation (i.e., those unable to attend school due to mental disability), those who had epilepsy or schizophrenia, or those who were participating in a different study that included use of a treatment intervention or investigational drug.

Data collection for the study occurred during visits within the normal course of care. Baseline data were collected from the routine outpatient visit in which patients were enrolled. Subsequent data collection was targeted at 1 month after the baseline visit and then every 3 months (3, 6, 9, and 12 months) after the baseline visit. Patient demographics and clinical history were recorded at the baseline assessment. Clinical severity of ADHD symptoms were assessed by treating physicians at each visit using the Clinical Global Impressions-ADHD-Severity (CGI-ADHD-S) scale [23] and Category A of the Child Symptom Inventory-4 Parent Checklist (CSI-4) [24]. The physician-rated version, of which CSI-4 scores were recorded by the treating physicians in consultation with parents, was used after converting scores to norm-referenced standardised CSI-4 scores using U.S.-based population norms.

### Treatment cohorts

Patients could be prescribed any treatment regimen by the treating physician. Treatment decisions were made solely at the discretion of the physician, patient, or parent/guardian and were independent of study participation. In actual practice, patients could receive no treatment/intervention, psychotherapy, pharmacotherapy, a combination of psychotherapy and pharmacotherapy, or other treatments. The most commonly prescribed medication at baseline was methylphenidate (45.5% [n = 486/1,068] of the total sample; 69.0% [n = 486/704] of patients who were prescribed at least one medication). Psychotherapy included formal sessions of psychoeducation and counselling, cognitive behavioural therapy, hypnotherapy, family therapy, or psychodynamic therapy that were conducted by a certified healthcare provider at a regular frequency for an acceptable length of time. Other treatments included educational interventions, speech therapy, occupational therapy, herbal therapy/homeopathy, informal hypnosis, psychomotor/physiotherapy, electroencephalogram biofeedback, diet exclusion, diet supplementation, and relaxation techniques. Further details on treatment patterns are available elsewhere [16].

Patients were categorised into two groups for the current analysis. The no treatment group included patients who received no ADHD treatments/interventions and patients who received other treatments at baseline. The treatment group included patients who were prescribed pharmacotherapy, psychotherapy, or both at baseline. As shown in Table 1, all three treatment groups (i.e., psychotherapy only, pharmacotherapy only, and both) had higher levels of clinical severity than the two no treatment groups (i.e., no treatments/interventions and other treatments). The level of clinical severity among patients who initiated psychotherapy only was highest in East Asia but was similar to those who initiated pharmacotherapy with/without psychotherapy in Central Europe.

**Table 1:** **Mean (SD) patient age and clinical severity by type of treatments prescribed at baseline**

**Variables**	**No TX**	**Other TX**	**Psycho**	**Pharmaco**	**Combi**	**P value**
**All**						
Patient age	8.60 (2.49)	7.90 (2.14)	8.66 (2.06)	9.13 (2.61)	9.24 (2.51)	<0.001
CGI-ADHD	4.16 (1.01)	4.33 (0.94)	4.80 (1.12)	4.51 (0.93)	4.55 (1.01)	<0.001
CSI-4	72.63 (11.02)	73.58 (10.30)	76.31 (10.08)	75.29 (10.49)	78.65 (10.43)	<0.001
**East Asia only**						
Patient age	8.60 (2.66)	7.61 (1.98)	9.05 (2.15)	9.20 (2.58)	8.95 (2.41)	<0.001
CGI-ADHD-S	4.01 (0.86)	4.50 (0.95)	5.23 (0.90)	4.52 (0.93)	4.65 (0.99)	<0.001
CSI-4	71.21 (10.95)	73.22 (9.38)	76.25 (9.68)	74.61 (9.08)	73.33 (9.27)	0.022
**Central Europe only**						
Patient age	8.59 (2.32)	8.64 (2.38)	8.34 (1.95)	9.04 (2.66)	9.32 (2.54)	0.037
CGI-ADHD-S	4.30 (1.11)	3.91 (0.75)	4.46 (1.16)	4.49 (0.93)	4.53 (1.01)	0.041
CSI-4	73.99 (10.96)	74.50 (12.55)	76.35 (10.49)	76.25 (12.16)	80.14 (10.27)	<0.001

*Abbreviations*: *SD* standard deviation, *ADHD* attention-deficit/hyperactivity disorder, *CGI-ADHD-S* Clinical Global Impressions-ADHD-Severity, *CSI-4* Child Symptom Inventory-4 Parent Checklist, *TX* treatment, *Psycho* psychotherapy only, *Pharmaco* pharmacotherapy only, *Combi* the combination of both psychotherapy and pharmacotherapy.

### Statistical analysis

Mean patient age and clinical severity by different types of treatments or no treatments prescribed at baseline (i.e., no treatments/interventions, other treatments, psychotherapy only, pharmacotherapy only, and the combination of psychotherapy and pharmacotherapy) were first examined and compared using one-way analysis of variance. Baseline characteristics of the patients who did and did not initiate treatment (i.e., psychotherapy, pharmacotherapy, or both) at baseline were then summarised and compared using *t* tests (for numerical variables) and chi-square tests (for categorical variables).

Logistic regression analysis was performed to explore patient characteristics associated with treatment initiation at baseline. The model included patient age, gender, age at first symptoms, region (Central Europe or East Asia), birth problems, family history of ADHD, and CGI-ADHD-S score. The model also included the following variables, but only if they appeared to be significant at p < 0.1 in simple logistic regressions: body mass index (BMI) (kg/m^2^), having other children living at home, mother having paid employment, father having paid employment, emotional health problems of parents/guardians due to their children’s behavioural problems, having been truant in the 4 weeks before baseline, having been involved in bullying (as a bully) in the 4 weeks before baseline, having primary care visits for behavioural problems in the 4 weeks before baseline, having been invited to social activities in the 4 weeks before baseline, and CSI-4 score. All analyses were repeated for each region, with the same list of the variables included in the model for the whole sample. The interactions between each factor and region were also examined to check whether the behaviour of the factors in treatment initiation, statistically significantly, differs by region. Each interaction model included region, each specific factor, and their interaction term. It should however be noted that the statistical significance of these interaction terms could be more meaningfully interpreted when the factors exhibit different directions of associations with treatment initiation in the regional subgroup analyses. In this case, the p-values of the interaction terms can indicate whether the different associations observed between regions are indeed statistically significant. Otherwise, their interpretations could be less straightforward. For instance, their interaction terms could appear to be non-significant when the behaviour of the factors in treatment initiation is similar between regions but the characteristics of the factors themselves still differ by region.

Statistical analyses were conducted using SAS version 9.1 software [25].

## Results

### Patient characteristics

**Figure 1:**
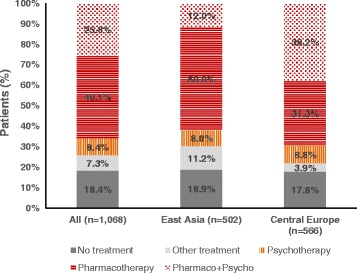
**Type of treatments prescribed at baseline.** Note that patients were categorised into two cohorts (no treatment and treatment) for the current analysis. The former includes patients who received no treatment and other treatment, and the latter includes patients who received pharmacotherapy and/or psychotherapy at baseline.

A total of 1,068 paediatric patients newly diagnosed with ADHD symptoms were included in this analysis (n = 566 in Central Europe and n = 502 in East Asia). Approximately three-quarters of these patients (74.3%, n = 794) initiated pharmacotherapy and/or psychotherapy at baseline (8.4% for psychotherapy only, 40.1% for pharmacotherapy only, and 25.8% for both) (Figure 1). The rate of treatment initiation was higher in Central Europe (78.3%) than in East Asia (69.9%) (p = 0.002). This difference was mainly attributable to a higher rate of other treatments prescribed in East Asia (11.2% in East Asia and 3.9% in Central Europe) (Figure 1). Another notable difference between the two regions was a higher prescription rate for combined pharmacotherapy and psychotherapy in Central Europe (48.8%) than in East Asia (17.1%).

Table 1 summarises mean (standard deviation [SD]) patient age and mean (SD) clinical severity for the five treatment types prescribed at baseline in each region. In East Asia, the mean level of clinical severity was highest in patients who initiated psychotherapy only (CGI-ADHD-S, 5.23 [SD 0.90]; CSI-4, 76.25 [SD 9.68]), whereas it was lowest in a subgroup of patients who did not initiate any treatments/interventions (CGI-ADHD-S, 4.01 [SD 0.86]; CSI-4, 71.21 [SD 10.95]). Mean patient age was lowest in those with other treatments (7.61 years [SD 1.98]), followed by those with no treatments/interventions (8.60 [SD 2.66]). In Central Europe, the mean level of clinical severity was highest in a subgroup of patients who initiated combination psychotherapy and pharmacotherapy (CGI-ADHD-S, 4.53 [SD 1.01]; CSI-4, 80.14 [SD 10.27]) and lowest in those who initiated other treatments or no treatments/interventions. Mean age was also highest in the combination group (9.32 [SD 2.54]) but lowest in the psychotherapy only group (8.34 [SD 1.95]).

The baseline patient characteristics by treatment initiation (i.e., psychotherapy and/or pharmacotherapy) are reported in Table 2. Patients who initiated treatment at baseline were more likely to be living in Central Europe, have had birth problems, have had primary care visits for behavioural problems in the 4 weeks before baseline, been truant in the 4 weeks before baseline, have parents/guardians experiencing emotional difficulties due to their child’s behavioural problems, have mothers not in paid employment, and have been invited to social activities in the 4 weeks before baseline. In addition, these patients were older and had greater mean clinical severity (i.e., higher CGI-ADHD-S and CSI-4 scores).

**Table 2:** **Baseline patient characteristics by treatment initiation at baseline**

**Baseline characteristic**	**No treatment (n = 274)**	**Treatment (n = 794)**
Age, mean (SD)*	8.40 (2.41)	9.12 (2.52)
Male, %	81.68	82.38
Age at first symptoms, mean (SD)	5.50 (2.43)	5.49 (2.33)
Being from East Asia (vs. Central Europe), %*	55.11	44.21
BMI (kg/m2), mean (SD)	18.01 (3.49)	18.31 (3.64)
Birth problems, %*	19.35	29.91
Family history of ADHD, %	40.20	47.10
Having other children living at home, %	54.25	57.47
CGI-ADHD-S, mean (SD)*	4.21 (0.99)	4.56 (0.98)
CSI-4 Category A (standardised), mean (SD)*	72.90 (10.80)	76.55 (10.53)
Paid employment (mother), %*	78.13	69.01
Paid employment (father), %	89.45	87.32
Parental psychological distress due to their children’s behavioural problems, %*	38.15	51.26
Being truant in the 4 weeks before baseline, %*	4.18	8.75
Being involved in bullying (as a bully) in the 4 weeks before baseline, %	25.58	22.68
Primary care visit for behavioural problems in the 4 weeks before baseline, %*	7.42	14.08
Being invited to social activity in the 4 weeks before baseline, %*	48.57	55.95

*Significant at p < 0.05 *Abbreviations*: *BMI* body mass index, *ADHD* attention-deficit/hyperactivity disorder, *CGI-ADHD-S* Clinical Global Impressions-ADHD-Severity, *CSI-4* Child Symptom Inventory-4 Parent Checklist, *SD* standard deviation.

### Patient characteristics associated with treatment initiation at baseline

Table 3 shows the results of logistic regression, which examined patients’ baseline characteristics associated with treatment initiation. At baseline, patients who initiated treatment tended to be older, have had birth problems, and have higher CGI-ADHD-S and CSI-4 scores, whereas those who did not initiate treatment tended to have been involved in bullying (as a bully) and have a working mother.

**Table 3:** **Factors associated with treatment initiation at baseline**

	**All**			**East Asia only**			**Central Europe only**			**Interaction with region** ^**‡**^
**Baseline factors**	**OR** ^**†**^	**95% CI**	**P-value**	**OR** ^**†**^	**95% CI**	**P-value**	**OR** ^**†**^	**95% CI**	**P-value**	**P-value**
Age	1.14	1.04, 1.24	0.005*	1.17	1.04, 1.31	0.009*	1.04	0.89, 1.21	0.599	0.227
Age at first symptoms	1.00	0.91, 1.10	0.995	0.97	0.85, 1.10	0.633	1.02	0.87, 1.19	0.835	0.622
Being male	1.24	0.74, 2.07	0.421	1.95	1.02, 3.73	0.043*	0.46	0.16, 1.33	0.154	0.034*
Being from East Asia	0.87	0.56, 1.36	0.533	-	-	-	-	-	-	-
Having birth problems	1.86	1.14, 3.02	0.013*	1.76	0.90, 3.45	0.099	1.74	0.79, 3.80	0.167	0.662
Family history of ADHD	1.08	0.71, 1.63	0.730	1.23	0.71, 2.14	0.463	0.94	0.47, 1.88	0.856	0.948
Higher CGI-ADHD-S scores	1.32	1.07, 1.63	0.010*	1.61	1.19, 2.17	0.002*	1.02	0.73, 1.43	0.915	0.185
Higher CSI-4 scores	1.03	1.01, 1.05	0.013*	1.02	0.99, 1.05	0.189	1.04	1.01, 1.07	0.022*	0.877
Having a working mother	0.60	0.38, 0.94	0.026*	0.68	0.38, 1.22	0.199	0.47	0.21, 1.05	0.066	0.291
Parental psychological distress	1.20	0.80, 1.81	0.376	0.64	0.38, 1.09	0.103	4.33	1.91, 9.81	<0.001*	<0.001*
Being truant^§^	1.47	0.56, 3.82	0.434	2.22	0.44, 11.14	0.334	1.83	0.49, 6.77	0.368	0.428
Being involved in bullying (as a bully)^§^	0.55	0.36, 0.85	0.007*	0.82	0.47, 1.44	0.491	0.27	0.13, 0.60	0.001*	0.674
Primary care visit for behavioural problems^§^	1.69	0.86, 3.34	0.130	1.90	0.81, 4.49	0.141	1.28	0.36, 4.61	0.702	0.912
Being invited to social activity^§^	1.23	0.83, 1.82	0.309	1.39	0.83, 2.33	0.216	1.27	0.64, 2.52	0.496	0.019*

^**†**^OR > 1 indicates a positive association with treatment initiation. The models included patient age, gender, age at first symptoms, region, birth problems, a family history of ADHD, and CGI-ADHD-S score. The rest of the variables were also included as they appeared to be significant at p < 0.1 in simple regressions with the whole sample. The same list of the variables were also included in the subgroup analyses by region, except for the variable “region”.

^‡^These p-values are for the interactions between region and each specific variable taken from the models that included region, each specific variable, and their “interaction” term.

^§^Behaviour in the 4 weeks before baseline.

*The asterisks (*) indicate p-values of <0.05.

*Abbreviations*: *ADHD* attention-deficit/hyperactivity disorder, *CGI-ADHD-S* Clinical Global Impressions-ADHD-Severity, *CSI-4* Child Symptom Inventory-4 Parent Checklist, *OR* odds ratio, *CI* confidence interval.

In East Asia, being older (odds ratio [OR] = 1.17; 95% confidence interval [CI], 1.04, 1.31; p = 0.009), being male (OR = 1.95; 95% CI 1.02, 3.73; p = 0.043), and having higher CGI-ADHD-S scores (OR = 1.61; 95% CI 1.19, 2.17; p = 0.002) were associated with treatment initiation. In Central Europe, parental psychological distress (OR = 4.33; 95% CI 1.91, 9.81; p < 0.001) and having higher CSI-4 scores (OR = 1.04; 95% CI 1.01, 1.07; p = 0.022) were positively associated with treatment initiation, whereas being involved in bullying (as a bully) (OR = 0.27; 95% CI 0.13, 0.60; p = 0.001) was inversely associated with treatment initiation at baseline. Notably, of these variables, being male and parental psychological distress exhibited different directions of odds ratios for treatment initiation in these regional subgroup analyses, and their interactions with region appeared to be statistically significant (0.034 for being male and <0.001 for parental psychological distress).

## Discussion

There is now high-quality evidence supporting the efficacy and effectiveness of a range of pharmacological treatments in the management of children and adolescents with ADHD [8,10]. In addition, a number of psychological interventions are also available as treatment options for ADHD, although their effects have been challenged by a recent meta-analysis limited to trials with “probably blind” assessment [26]. The findings of this large 1-year observational study, however, revealed that 25.7% of patients newly diagnosed with ADHD symptoms in Central Europe and East Asia did not initiate treatment (i.e., pharmacotherapy and/or psychotherapy) at baseline. This rate was higher in East Asia (30.1%) compared with Central Europe (21.7%), mainly due to a higher rate of other nonconventional treatments (e.g., herbal therapy) prescribed in East Asia. Nevertheless, clinical severity appeared to be one of the most important determinants of treatment initiation in both regions.

The role of clinical severity in treatment decision making may be even greater in East Asia than Central Europe. There were only three factors associated with treatment initiation in East Asia, which were being older, being male, and having higher clinical severity (measured by the CGI-ADHD-S). These three factors may indicate more severe clinical symptoms and a higher tolerability of drug treatments. Given the high acceptance of herbal treatments (traditional Chinese medicine) and high levels of stigma attached to mental illness in East Asia [15], it is possible that the initiation of formal treatment for ADHD is more conservative in this region, thereby its use is strictly limited to school-aged children and adolescents with severe clinical symptoms and impairments, as hypothesised.

However, it is noteworthy that the role of clinical severity or patient age does not appear to be obvious when making the decision between pharmacotherapy and psychotherapy in actual clinical practice in East Asia. Level of clinical severity was highest in a subgroup of patients who initiated psychotherapy only. This is somewhat inconsistent with international guidelines, which generally recommend the use of psychotherapy for milder ADHD and for younger children [8,10,11]. This might be partly due to the composition of our patient sample, who were patients newly diagnosed with ADHD symptoms, although the same pattern was not observed in Central Europe. Alternatively, this could reflect negative attitudes toward the use of medications for ADHD in East Asia, which might be much stronger there than in other parts of the world [27].

Clinical severity was also associated with treatment initiation at the baseline visit in Central Europe. Although it was highest in the subgroup of patients who initiated both psychotherapy and pharmacotherapy, 48.8% of patients started with the combination treatment at baseline, despite the fact that patients were all “newly” diagnosed with ADHD; this rate is much higher than that for East Asia (17.1%). This is another geographical discrepancy in treatment practices that needs to be further investigated.

In addition to clinical severity, parental psychological distress due to their child’s behavioural problems was also strongly associated with treatment initiation in this region. Although this variable could, in part, reflect the level of clinical severity of their affected child, it is not clear why this relationship was not observed in East Asia. In fact, parental psychological distress was inversely associated with treatment initiation in East Asia, although the association was not statistically significant (OR = 0.64; 95% CI 0.38 – 1.09; p = 0.103). The interaction between region and parental psychological distress, however, still appeared to be statistically significant (p < 0.001). Interestingly, previous research using the same data found that parental psychological distress was associated with medication nonadherence during follow-up in East Asia but not in Central Europe [20]. The Taiwanese study by Gau *et al*. also reported similar findings [28].

These seemingly contradictory findings may imply that different mechanisms underlie the impacts and causes of parental psychological distress in these regions. In Central Europe, parental psychological distress may indicate a greater need for professional help on their affected child and could be one of the main determinants of the decision to seek help, whereas in East Asia, the impacts and causes of parental psychological distress could be more complex. Many parents of children and adolescents with ADHD in East Asia tend to take personal responsibility for their child’s behavioural problems and often have negative attitudes toward the use of medications for ADHD [27]. In East Asia, parents who also take heavy responsibility for their child’s problems and extensively intervene to mediate behaviour are also more likely to be those who have psychological distress caused by their child’s behavioural problems. It is possible that these types of parents are more conservative in the use of medication for their child’s ADHD symptoms and are willing to initiate treatments only when their child’s symptoms are sufficiently severe and cannot be appropriately controlled by their own interventions. They may also stop such treatments more easily once their child’s symptoms improve, potentially leading to a higher level of medication nonadherence in East Asia. Further research, culturally sensitive, is required to better understand the role of parental psychological distress in the treatment of ADHD.

Our study also found an inverse association between being involved in bullying (as a bully) and treatment initiation at baseline in Central Europe. It should be noted that a similar association was also observed in East Asia, but it was not statistically significant (OR = 0.82; 95% CI 0.47-1.44; p = 0.491). Although the reason for this inverse association is not clear, it is possible that given a higher possibility of other comorbid conditions among these patients (e.g., conduct disorder), clinicians could have needed more time to observe their symptoms before making a correct diagnosis and subsequent treatment plans. In addition, the use of stimulants, which were the only medications (or dominant medications) available for the treatment of ADHD in many countries in Central Europe and East Asia at the time this observational study was conducted, might have been discouraged for these children as first-line therapy due to potential risks of drug abuse or diversion.

However, several limitations of this study should be taken into account when interpreting these results. Firstly, given its observational design, the associations found in our study do not imply causal relationships. Secondly, the patients included in our study were diagnosed with the symptoms of ADHD, not necessarily with ADHD itself. The broader inclusion criteria were designed to observe and describe the similarities and differences in actual treatment practices and prescription patterns across several countries in Central Europe and East Asia. Although this is not a limitation in the context of our study, the role of clinical severity in treatment decision making could have been greater with such patients because they are potentially more heterogeneous in terms of symptom severity. Thirdly, the treatment initiation can also be influenced by the socioeconomic status of patients (e.g., income, insurance), especially in Central Europe and East Asia, where a high level of patient cost-sharing is common. However, due to data constraints, our study was unable to assess their impact on treatment initiation. Finally, although this observational study included more than one thousand patients, they may not be representative of the paediatric patients with ADHD symptoms in Central Europe and East Asia.

## Conclusions

Our findings indicate systematic differences in patient characteristics between patients who did and did not initiate treatment (pharmacotherapy and/or psychotherapy) at baseline in the treatment of ADHD symptoms. Although factors associated with treatment initiation differed to some extent between Central Europe and East Asia, clinical severity appeared to be one of the most important determinants of treatment initiation in both regions but was possibly more so in East Asia. However, the choice between pharmacotherapy and psychotherapy, either alone or in combination, varied substantially across the regions.

## Additional file

Additional file 1:
**A list of all ethical review boards that approved the B4Z-VI-B004 observational study.**
