# Supplementary material for: Patient characteristics associated with treatment initiation among paediatric patients with Attention-Deficit/Hyperactivity Disorder symptoms in a naturalistic setting in Central Europe and East Asia
Source: BMC Psychiatry. 2014 Oct 30;14:304. doi: 10.1186/s12888-014-0304-x (PMC4219015; doi:10.1186/s12888-014-0304-x)
Supplement: Additional file 1: — A list of all ethical review boards that approved the B4Z-VI-B004 observational study. [file 12888_2014_304_MOESM1_ESM.docx]

**A list of all Ethical Review Boards that approved the B4Z-VI-B004 observational study**

**China**

Ethical committee of Shanghai Mental Health Center, Shanghai

Ethical committee of Shanghai Changzheng Hospital, Shanghai

Ethical committee of Shanghai Children’s Medical Center, Xin Hua Hospital, Shanghai

Ethical committee of the 6^th^ Affiliated Hospital of peking University, Beijing

Ethical committee of Beijing Anding Hospital, Beijing

Ethical committee of THE CAPITAL Institute of Pediatrics, Beijing

Ethical committee of Beijing Children’s Hospital, Beijing

Ethical committee of The Third Affiliated Hospital of Sun Yat-sen University, Guangzhou

**The Czech Republic**

Eticka komise Krajske nemocnice Liberec, Liberec

**Hungary**

OPNI Klinikopharmakológiai Kutatásetikai Bizottsága, Budapest

Főv. Önk. Bud. Gyermekkórház Kórházi Etikai Bizottság, Budapest

SE Egészségtudományi Kar, Tudományos és Kutatásetikai Bizottsága, Budapest

Pécs Mj. Városi Egyesített Egészségügyi Intézmények Etikai Bizottsága, Pécs

SzTE Regionális Humán Orvosbiológiai Kutatásetikai Bizottság, Szeged

Pándy Kálmán Kórház Humán Kutatásetikai Bizottsága, Gyula

HBM-i Kenézy Gyula Kórház-Rendelőintézet Intézeti Kutatásetikai Bizottsága, Debrecen

**Romania**

Comisia NAŢIONALĂ DE ETICĂ, BUCUREŞTI

**Slovak Republic**

Eticka komisia Psychiatrickej nemocnice Michalovce, Michalovce

**South Korea**

Seoul National University Hospital Ethical Review Board, SEOUL

Hanyang University Hospital Ethical Review Board, SEOUL

Gangnam Severance Hospital Ethical Review Board, SEOUL

Asan Medical Center Ethical Review Board, SEOUL

Chungang University Hospital Ethical Review Board, SEOUL

**Taiwan**

National Taiwan University Hospital Research Ethics Committee, taipei

Chang Gung Medical Foundation Institutional Review Board, taipei

**Turkey**

Dokuz Eylul University Faculty of Medicine Ethical Review Board, Izmir

Gaziantep University Faculty of Medicine Ethical Review Board, Gaziantep

Ankara University Faculty of Medicine Ethical Review Board, Ankara

Mersin University Faculty of Medicine Ethical Review Board, Mersin

Hacettepe University Faculty of Medicine Ethical Review Board, Ankara

Uludag University Faculty of Medicine Ethical Review Board, bursa
